# Far field superfocusing along with enhanced near field emission from hybrid spiral plasmonic lens inscribed with nano corrals slit diffractor

**DOI:** 10.1038/s41598-018-19571-z

**Published:** 2018-01-18

**Authors:** Priyanshu Jain, Tanmoy Maiti

**Affiliations:** Department of Materials Science and Engineering, Plasmonics and Perovskites Laboratory, Indian Institute of Technology Kanpur, UP, 208016 India

## Abstract

Here, we have numerically calculated electric field intensity and phase of the emission from various hybrid spiral plasmonic lenses (HSPL) in near field as well as in far-field. We have proposed a novel HSPL inscribed with nano corrals slit (NCS) and compared its focusing ability with other HSPLs inscribed with circular slit and circular grating. With the use of nano corrals slit, we have been able to improve light intensity in the far-field without compromising near-field intensity. Our NCS-HSPL outperforms other HSPLs and standalone SPL in near-field as well as far-field. We have also found that proposed circular slit diffractor is far more superior than previously reported circular grating diffractor. We have been able to extend the focal length of hybrid plasmonic lens upto 3 um and observed a two-fold increment in the far field intensity compared to existing spiral plasmonic lens even though size of focal spot remains same. Optical complex fields produced by NCS based HSPL can be used for various applications such as super resolution microscopy, nanolithography, bioimaging and sensing, angular momentum detectors, etc. Moreover, enhanced near-field intensity in conjunction with far-field superfocusing with reasonable focal length may lead to the development of novel multifunctional lab-on-chip devices.

## Introduction

Surface plasmon polaritons (SPPs) are evanescent electromagnetic wave that propagates along metal-dielectric interface^1,2^. Having focusing ability in sub-wavelength scale coupled with stronger field enhancement effect of light, SPPs can be focused into tightly curbed spot with subwavelength size beyond the diffraction limit. Consequently, they have shown potentials in various applications such as plasmonic waveguide^3,4^, nanolithography^5,6^, nano-optics^7^, super-resolution imaging^8,9^, near-field imaging and sensing^10,11^, optical data storage^12,13^, etc. Focusing of SPPs by plasmonic lens i.e. circular/elliptical structure milled into optically thick metallic films on glass substrate has been experimentally demonstrated^14^. When the lens is excited by light, the incoming wave couples with SPPs originated in the metal/dielectric interface from the annular slit and forms a focal spot at the exit surface. Further, efforts have been made to achieve optical plasmonic focusing by varying geometries of plasmonic lens structure and implementing suitable polarization of incident beam^15,16^. Among the various plasmonic geometries, Archimedes spiral plasmonic lenses (ASPL) have been effective to focus SPPs at their center owing to the constructive interference between counter-propagating SPP waves generated from the spiral slit^17,18^.

Due to the evanescent nature of SPPs, their nano-focusing in the Spiral Plasmonic Lens (SPL) is always limited within the near field, which in turn restricts its possibilities for actual device fabrication, since most of them would require a good working distance from the surface of lens. Consequently, in order to increase the focal point of the plasmonic lens, various hybrid plasmonic lenses (HPL) have been proposed such as chirped circular nanoslits^19^, SPL with a concentric groove^20^, SPL with multi circular groove^21^. It has been observed that circular grooves have negligible effect on FWHM of the focal spot. Recently, SPL with central aperture antenna has been introduced describing relationship between the near field and far field distribution of the plasmonic structure^22^. It has been shown that focal spot position can be attuned by adjusting the incident angle of light. However, in all HPLs reported in the literature, improvement of the focal point in far field is achieved at the expense of electric field intensity in the near field. Moreover, role of linearly polarized light in far field focusing has rarely been discussed, and phase distribution of electric field in the far field has also been barely studied.

**Figure 1:**
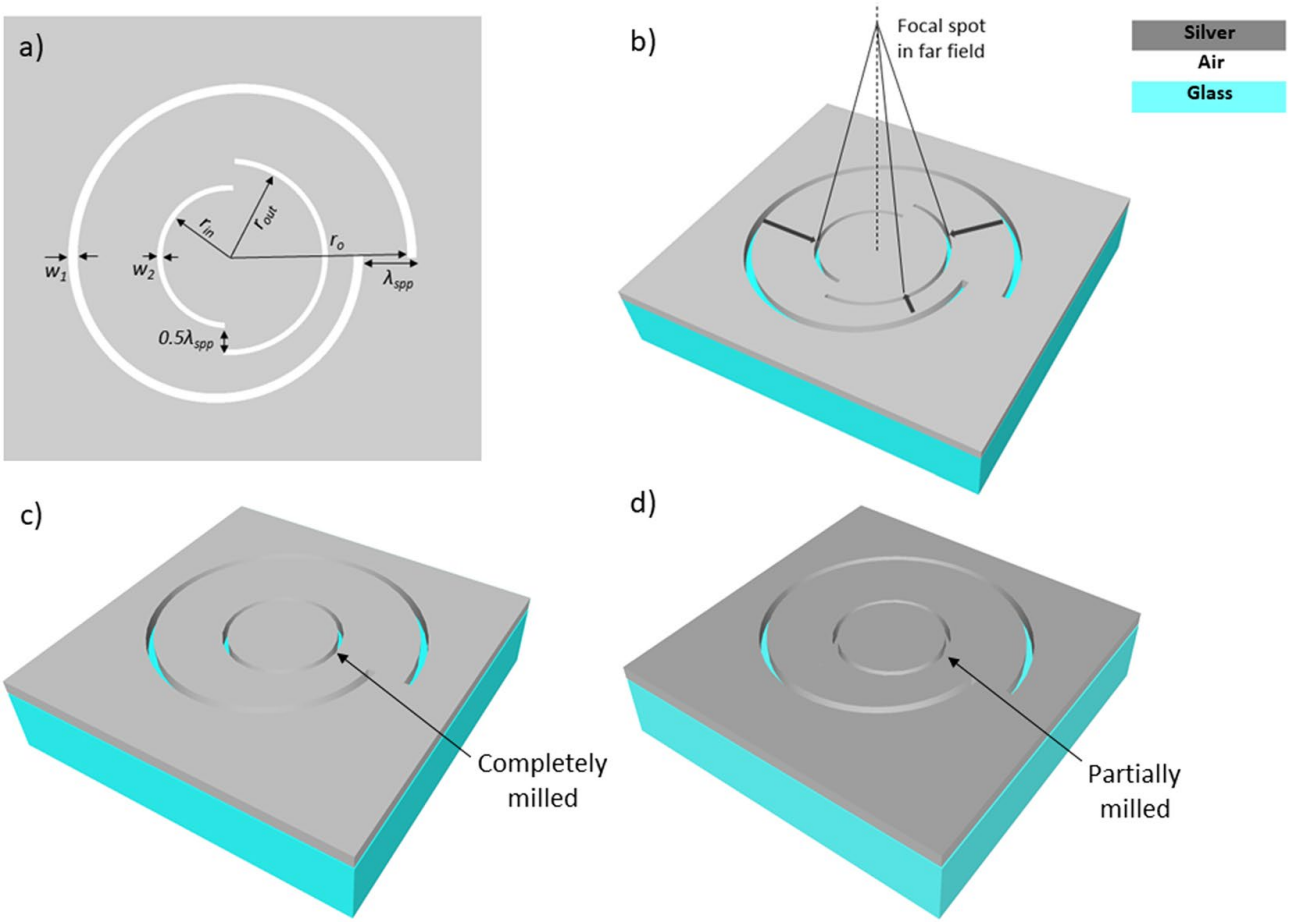
Schematic of proposed hybrid lens inscribing NCS (nano corrals slit) in SPL. (**a**) Top view of NCS lens and (**b**) 3D view of lens where arrow shows the direction of propagation of SPPs. (**c**) and (**d**) represents schematic of CPL (circular plasmonic lens) and CG (circular grating) in SPL respectively.

In the present work, we have proposed a novel plasmonic nanostructure that is constructed by inscribing nano corrals slit (NCS) within a conventional Archimedes SPL as shown in Fig. 1(a,b). Using our novel structure, we have been able to increase the focal point without compromising the overall intensity of Electric field at exit surface. NCS acts as a far field diffractor by converging the propagating SPP waves scattered from the slit and helps in generating a bright focal spot away from the surface of the lens. Along with NCS, we have inscribed previously reported^20,21^ circular grating (CG) inside SPL formed by partially milling a circular groove into the metal film as shown in Fig. 1d. Additionally, we have also inscribed circular plasmonic lens (CPL) inside SPL, which is formed by milling a circular groove all the way through the metal film as shown in Fig. 1c. We have compared and studied the focusing ability of these hybrid lenses in the near field as well as far field with standalone SPL. We have employed 3D FDTD simulation method to compute SPP’s electric field intensity and phase profile under both linearly and circularly polarized light. Plasmonic device architecture of the proposed novel plasmonic structure has been optimized by varying radius of the NCS diffractor. We have numerically calculated the intensity distributions in these hybrid lenses and attained a bright intensity focal spot in the far field. We have found that hybrid spiral plasmonic lens (HSPL) inscribing circular slit always performs better than HSPL inscribing circular grating in both near as well as far field. Simulation results have shown that our novel NCS-HSPL outperforms all other HSPLs in both near-field as well as far-field. Light intensity of the focal spot increases about twofold at 3 um above plasmonic lens surface with the use of NCS-HSPL, while the FWHM of the focal spot remains same size. Remarkably, we have been able to maintain the phase distribution of ASPL in the NCS-HSPL as well even after doubling the intensity of focal spot in the far field. Such a lens with long focal length can be used for many novel applications such as super resolution microscopy^23,24^, nano lithography^25,26^, bioimaging^27,28^, quantum communications^29,30^, angular momentum detectors^31,32^, etc. Moreover, these HSPLs which can focus with enhanced intensity at the near-field as well as far-field can pave the way of developing novel multifunctional devices for sensing and imaging.

## Design principle

Figure 1 shows the schematic of novel HSPL where NCS nanostructure is inscribed at the center of Archimedes SPL. NCS is formed by milling two semicircular, non-Archimedean spirals, of opposite spin concentrically into a silver film. The SPP waves excited by illuminating the back of the glass substrate at the silver-air interface propagate inward towards the center of the lens. The nano corrals slit are fabricated inside the spiral slit in anticipation that it will scatter the SPPs waves and convert them into propagating waves in free space as shown in Fig. 1 (3D-view). These propagating waves will interfere constructively on the optical axis and generate a single solid focal spot with bright intensity in the far field. Length of focal spot along the optical axis can be changed by varying the radius of NCS diffractor.

For the spiral slit aperture, width w_1_ is kept at 200 nm and the distance r from center can be expressed as *r* = *r*_*o*_ − (*λ*_*spp*_)*θ*/(2*π*) where r_o_, inner radius of SPL, is 1.6 um, λ_spp_ is the effective wavelength of SPP and *θ* is the azimuthal angle. Difference between inner and outer radius of spiral slit is kept at λ_spp_ in order to keep the difference of distance travelled by counter propagating SPP waves from the antipodal points equal to 0.5 λ_spp_. For nano corrals slit, width w_2_ is 150 nm and the difference of inner and outer radius is set at 0.5λ_spp_ to maintain the condition for constructive interference of SPPs at the center.

## Simulation Result and Discussion

In the present work, we have investigated three different HSPL by inscribing nano corrals slit (NCS), circular plasmonic lens (CPL) and circular grating (CG) inside ASPL. We have compared the focusing ability of these hybrid lenses in the near-field as well as far-field with standalone ASPL. Three-dimensional finite difference time domain (FDTD) simulations have been carried out to examine the influence of geometry of various plasmonic nano-structures and incident light polarization on the focusing properties. We have used illumination wavelength 415 nm and the corresponding surface plasmon wavelength at silver-air interface is 374 nm. A nano corrals slit structure inscribed concentrically inside Archimedes spiral slit is etched through a 150 nm silver film deposited on a glass substrate (*n* = 1.4) as shown in Fig. 1.

**Figure 2:**
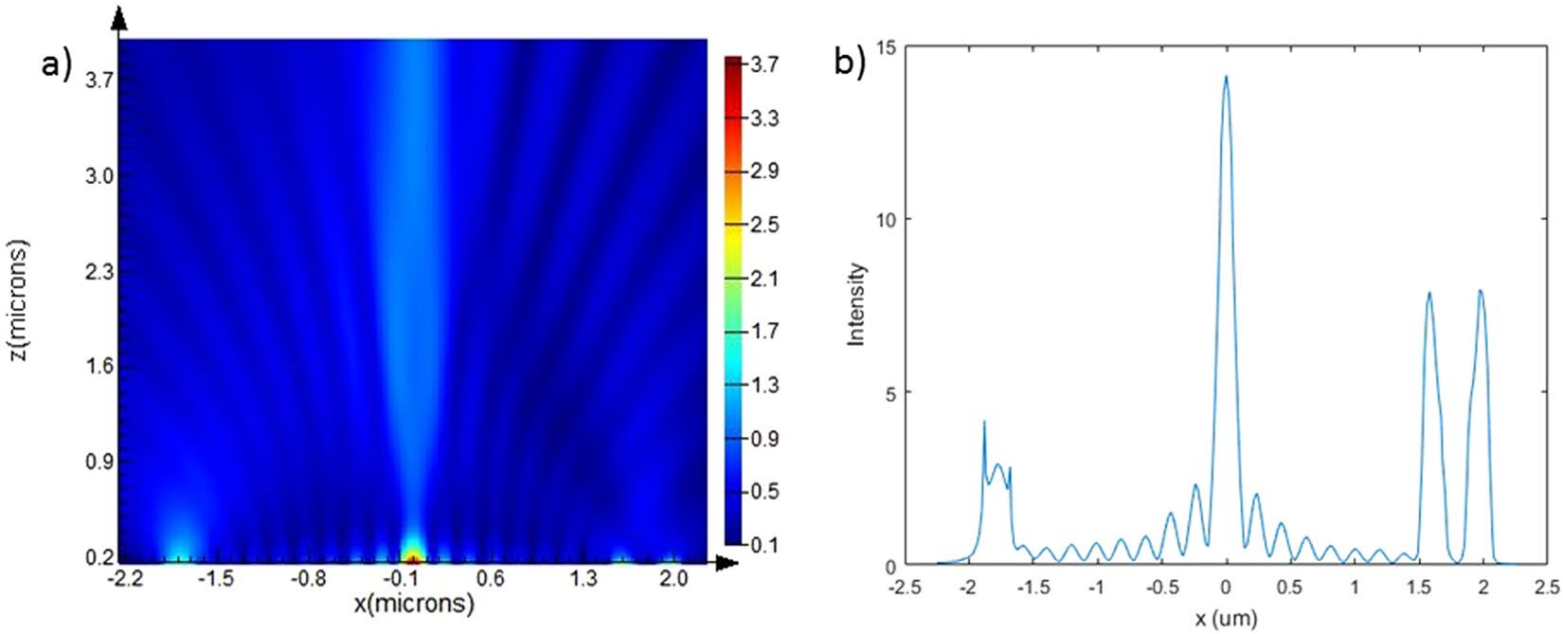
(**a**) Intensity distributions of SPL in XZ plane under RCP illumination. (**b**) Plot of intensity along x-axis at the surface of the lens.

Following above design, ASPL is excited with the RCP illumination from the back of the substrate. SPPs excited from the spiral slit propagate on the surface of the lens and forms bright spot at the center owing to the constructive interference of SPPs. The electric field intensity will have zero order Bessel function distribution at the exit surface as shown in Fig. 2a. The intensity of this distribution reaches maxima at the center of exit surface and a bright focal spot is developed in the near field.

A series of intensity minima can be observed at 0.14, 0.34, 0.52, 0.72, 0.90 um and so on from the center of the lens as shown in Fig. 2(b). Similarly, a series of intensity maxima can be observed at 0.23, 0.43, 0.61, 0.81, 0.99 and so on. It has been reported in the literature that in case of HSPL with circular grating (CG) as diffractor, when the groove is placed at these locations of minima an intensity maxima would emerge on the optical axis multiple wavelengths away from the exit surface of lens^20^. In the present work, we have compared the effect of radius of diffractor by placing nano corrals slit, circular groove and circular grating at these positions of minima as well as maxima.

**Figure 3:**
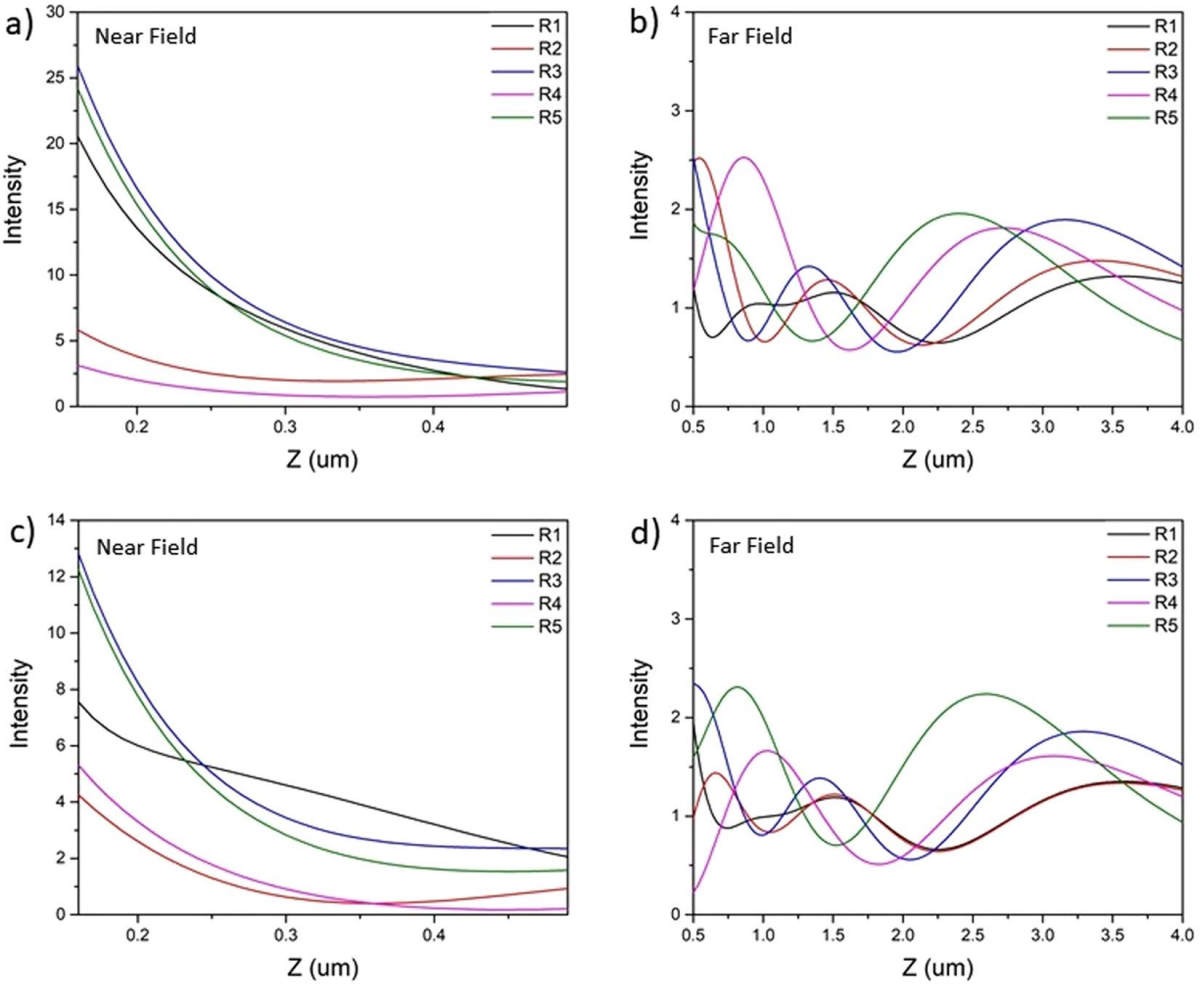
Intensity distributions on the optical axis for hybrid lens with NCS under RCP illumination. (**a**) and (**b**) represents NCS with slit radius R1 = 0.23, R2 = 0.43, R3 = 0.61, R4 = 0.81 and R5 = 0.99 um (intensity maxima position) for near and far field respectively. Similarly, (**c**) and (**d**) represents NCS with slit radius R1 = 0.14, R2 = 0.34, R3 = 0.52, R4 = 0.72 and R5 = 0.90 um (intensity minima position) for near and far field respectively.

We have varied the radius of NCS with respect to the maximum as well as minimum intensity positions of SPL (as shown in Fig. 2) and numerically computed the electric field’s intensity along z-axis in near field as well as far field is shown in Fig. 3. From Fig. 3(a,b) which corresponds to intensity maxima position of SPL, it is evident that NCS with inner radius of 0.61um (=R3) performs best in the near-field. Although NCS having radius of 0.61um (R3) and 0.99 um (R5) produce similar intensity values in the far-field, NCS with 0.61 um radius shows the maximum intensity at higher Z resulting the longer focal length, which favors it over others. However, when NCS is set at intensity minima position the one with radius of 0.52 um (=R3) performs better in near field but fails to deliver in far field and the one with radius of 0.90 um (=R5) performs much better in far field but slightly underperforms in near field as shown in Fig. 3(c,d). Moreover, maximum intensity obtained in the far-field for NCS diffractors placed in the intensity minima position of SPL, is very similar to what we obtained for the best-performed NCS placed in the intensity maxima position of SPL. It suggests that as long as we place the NCS diffractor in the maxima or minima intensity position of SPL we can achieve good intensity in the far-field at optimized inner radius of NCS. However NCS with inner radius of 0.61 um placed at the intensity maximum position in SPL exhibits much higher (twice) near field intensity compared to what can be obtained for NCS placed in the intensity minima positions of SPL besides keeping up the similar maximum intensity values in the far-field.

Therefore, we have chosen inner radius of NCS to be 0.61um since it outperforms all other radius combinations in both far field and near field. To make a meaningful comparison with NCS diffractor, radius of CPL and CG diffractors have also been kept at 0.61um. However, we performed similar studies on the effect of radius of CPL and CG diffractors in the far-field as well as in the near field as shown in Figs S5 and S6, respectively. It is observed that maximum intensity obtained in the far-field for earlier reported CG diffractor is almost half of the intensity value obtained in the NCS based HSPL. However our proposed CPL diffractor produces maximum intensity values in the far-field similar to NCS.

According to above analysis, specifications of the plasmonic nano structure shown are optimized as follows:

Inner radius of the spiral slit, r_o_ = 1.6 ums

Width of the spiral slit, w_1_ = 200 nm

Width of nano corrals slit, w_2_ = 200 nm

Smaller radius of nano corrals slit, r_in_ = 0.6159 um

Larger radius of nano corrals slit, r_out_ = 0.8029 um

Thickness of all hybrid lenses are 150 nm except circular grating which has a thickness of 100 nm. Radii difference of NCS is kept such that counter propagating SPPs are in phase when they arrive at the center. Counter propagating SPPs generated at the diametrically opposite points on the slit surface already have a phase difference of π. In order to get a bright spot at the center we should induce an additional phase difference of π by adjusting the pitch length. Phase difference at the center of NCS due to waves arriving from diametrically opposite points at can be written as k_spp_ (*r*_*out*_ − *r*_*in*_). This should be odd multiple of π.

∴ k_spp_ (*r*_*out*_ − *r*_*in*_) = *π* and hence (*r*_*out*_ − *r*_*in*_) = *λ*_spp_/2. Thus we have kept pitch length of NCS at 0.5 λ_spp_.

**Figure 4:**
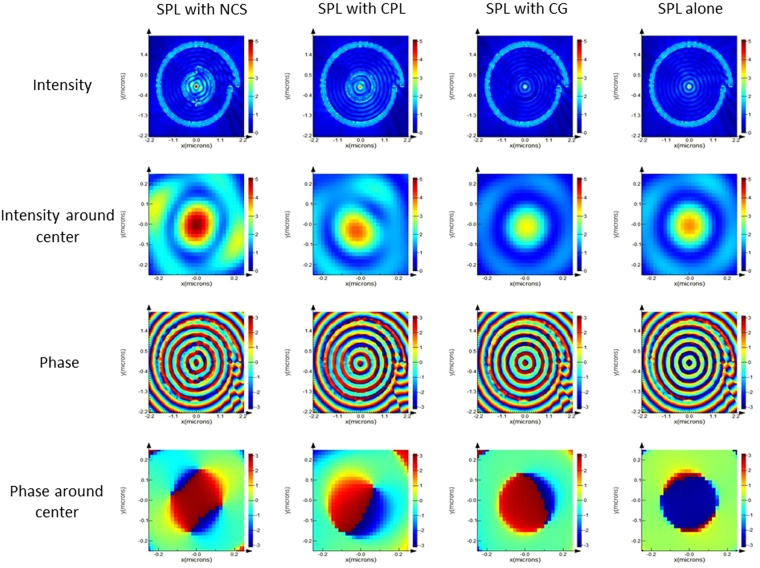
RCP illumination on HSPL: FDTD simulation results of various diffractors inscribed within SPL under RCP illumination in near field. 1^st^ and 3^rd^ row represents intensity and phase profile of lens while 2^nd^ and 4^th^ row represents intensity and phase profile at the center of the lens.

**Figure 5:**
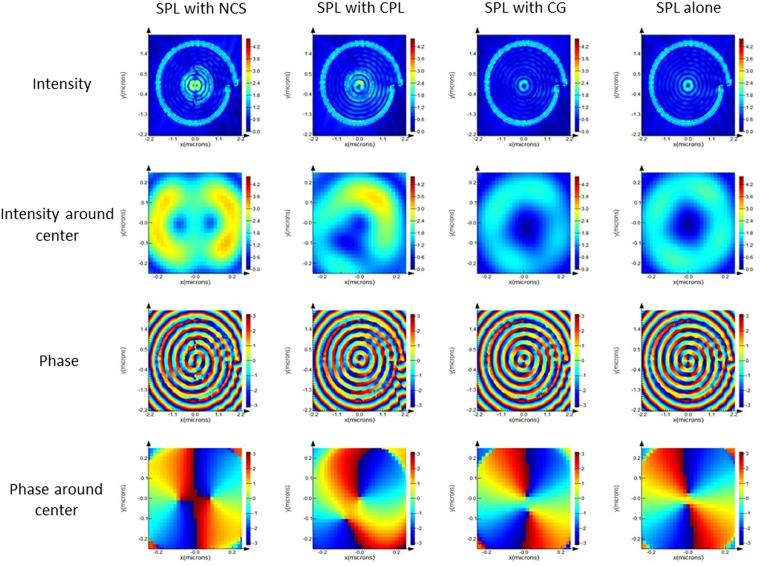
LCP illumination on HSPL: FDTD simulation results of various diffractors inscribed within SPL under LCP illumination in near field. 1^st^ and 3^rd^ row represents intensity and phase profile of lens while 2^nd^ and 4^th^ row represents intensity and phase profile at the center of the lens.

FDTD simulation in the near field has been carried out to study the effect of polarization of incident light on the electric field distribution and phase distribution of various hybrid lenses. Figures 4 and 5 shows simulation results of the near-field intensity and phase distribution for HSPL inscribing NCS, CPL and CG diffractor along with standalone SPL under RCP and LCP illumination respectively. Under RCP illumination as shown in Fig. 4 (1^st^ column) NCS diffractor produces bright spot at the center of the lens with highest intensity among all HSPLs, followed by CPL diffractor, simple SPL and CG diffractor. These lenses exhibit similar trend in near-field intensity under x-polarized illumination as shown in Fig. S1. We have also observed that electric field intensity distribution of NCS-HSPL is similar to SPL under both RCP and x-polarized illumination with magnitude of electric field being enhanced inside NCS-HSPL. This enhancement effect is attributed to the contribution of NCS to the total electric field intensity of the lens. Standalone NCS plasmonic lens with pitch length 0.5λ_spp_ generates bright spot at the center under RCP light as shown in Fig. S2(a,b). This can be shown analytically using following equation^33^:

1 $${E}_{z}(\rho ,\theta ,z)={e}^{-{k}_{a}z}{\int }_{0}^{2\pi }A(\varphi (\theta ^{\prime} )){e}^{j\omega (\varphi (\theta ^{\prime} ),\theta ^{\prime} )}{e}^{j{k}_{spp}|\rho -\rho ^{\prime} |}{d}{\theta }^{\prime} $$

where, θ, ρ, z indicates the azimuthal, radial, and z-directional coordinates for point of observation near the center of plasmonic lens and θ′, ρ′, z′ indicates the coordinates of dipole sources which are further integrated along periphery of lens. K_a_ denotes the extinction coefficient of SPP in air. The term |ρ−ρ′| represents distance between the source of plasmons and the point of investigation. The term |ρ−ρ′| in the equation 1 has been modified for NCS based on the geometry (Fig. S2(a)) as follows:

for *θ* = (−π/2, π/2): $|{\rm{\rho }}-{\rm{\rho }}^{\prime} |={r}_{o}+0.5{\lambda }_{spp}-{\rm{\rho }}\,\cos (\theta -\theta ^{\prime} )$; r_o_ is the radius of smaller semi-circle of NCS

for *θ* = (π/2, 3π/2): $$|{\rm{\rho }}-{\rm{\rho }}^{\prime} |={r}_{o}-{\rm{\rho }}\,\cos (\theta -\theta ^{\prime} )$$

for *θ* = (−π/2, π/2):$${e}^{j{k}_{spp}|\rho -\rho ^{\prime} |}={e}^{j{k}_{spp}.{r}_{o}}{e}^{j\pi }{e}^{-j{k}_{spp}.{\rm{\rho }}\cos (\theta -\theta ^{\prime} )}=-{e}^{j{k}_{spp}.{r}_{o}}{e}^{-j{k}_{spp}.{\rm{\rho }}\cos (\theta -\theta ^{\prime} )}$$

and for *θ* = (π/2, 3π/2): $${e}^{j{k}_{spp}|\rho -\rho ^{\prime} |}={e}^{j{k}_{spp}.{r}_{o}}{e}^{-j{k}_{spp}.{\rm{\rho }}\cos (\theta -\theta ^{\prime} )}$$

Now plugging all the values in equation 1, we have

$$\begin{array}{rcl}{E}_{z}(\rho ,\theta ,z) & = & -{e}^{-{k}_{a}z}{\int }_{-\pi /2}^{\pi /2}{A}_{o}{e}^{-j\theta ^{\prime} }{e}^{j{k}_{spp}.{r}_{o}}{e}^{-j{k}_{spp}.{\rm{\rho }}\cos (\theta -\theta ^{\prime} )}{d}\theta ^{\prime} \\  &  & +\,{e}^{-{k}_{a}z}{\int }_{\pi /2}^{3\pi /2}{A}_{o}{e}^{-j\theta ^{\prime} }{e}^{j{k}_{spp}.{r}_{o}}{e}^{-j{k}_{spp}.{\rm{\rho }}\cos (\theta -\theta ^{\prime} )}{d}\theta ^{\prime} \end{array}$$

Limits of the first integral term can be changed from (−*π*/2, *π*/2) to (*π*/2, 3*π*/2) with the proper variable substitution

2 $$\begin{array}{rcl}{E}_{z}(\rho ,\theta ,z) & = & {e}^{-{k}_{a}z}{\int }_{\pi /2}^{3\pi /2}{A}_{o}{e}^{-j\theta ^{\prime} }{e}^{j{k}_{spp}.{r}_{o}}{e}^{j{k}_{spp}.{\rm{\rho }}\cos (\theta -\theta ^{\prime} )}{d}\theta ^{\prime} \\  &  & +{e}^{-{k}_{a}z}{\int }_{\pi /2}^{3\pi /2}{A}_{o}{e}^{-j\theta ^{\prime} }{e}^{j{k}_{spp}.{r}_{o}}{e}^{-j{k}_{spp}.{\rm{\rho }}\cos (\theta -\theta ^{\prime} )}{d}\theta ^{\prime} \\ {E}_{z}(\rho ,\theta ,z) & = & {A}_{o}{e}^{-{k}_{a}z}{e}^{j{k}_{spp}.{r}_{o}}{\int }_{\pi /2}^{3\pi /2}{e}^{-j\theta ^{\prime} }(2\ast \,\cos ({k}_{spp}.{\rm{\rho }}\,\cos (\theta -\theta ^{\prime} ))){d}\theta ^{\prime} \\ \therefore \,\,{E}_{z}(\rho ,\theta ,z) & \propto  & {e}^{-{k}_{a}z}{\int }_{\pi /2}^{3\pi /2}{e}^{-j\theta ^{\prime} }\,\cos ({k}_{spp}.{\rm{\rho }}\,\cos (\theta -\theta ^{\prime} )){d}\theta ^{\prime} \end{array}$$

Normalized intensity based on above equation is plotted and shown in Fig. S2(c). It is evident from both simulation and analytical plot that under RCP illumination NCS produces bright spot. SPL itself also produces bright spot at the center under RCP illumination as per the following equation^34^:

3 $${E}_{z}(\rho ,\theta ,z)\propto {e}^{-{k}_{a}z}{J}_{0}({k}_{spp}\rho );$$

where J_o_ is zero-order Bessel’s function.

Therefore, total intensity at the center of the NCS-HSPL enhances due to cumulative intensity of NCS and SPL resulting a much brighter spot than SPL alone. However, CPL and CG individually creates dark spot at the center of the lens under RCP light and thus effectively adds nothing to the standalone SPL when we make HSPL. This can be seen from Fig. 4 (2^nd^ row) that NCS-HSPL has much brighter spot than SPL whereas CPL and CG-HSPL has comparable intensity with respect to SPL. Similar phenomenon happens when lenses are excited with x-polarized light where NCS creates bright spot at the center along with SPL. Hence, we have observed enhanced bright spot at the center while intensity magnitude in CPL and CG-HSPL remains same as standalone SPL as shown in Fig. S1 (2^nd^ row).

Under LCP illumination as shown in Fig. 5 (1^st^ column) NCS diffractor provides a bright intensity spot at the center of the lens while rest of the lenses have zero intensity at the center. Taking a closer magnified view in the NCS-HSPL it is evident that two dark spots with corresponding 1^st^ order vortex phase surrounding the light spot occur at the center. This particular case is very interesting since under LCP light SPL’s electric field intensity follows 2^nd^ order Bessel’s function i.e. dark spot at the center with 2^nd^ order vortex phase according to the following equation^34^:

4 $${E}_{z}(\rho ,\theta ,z)\propto {e}^{-{k}_{a}z}{e}^{2i\theta }{J}_{2}({k}_{spp}\rho );$$

where J_o_ is second order Bessel’s function.

In standalone CPL and CG electric field intensity has 1^st^ order Bessel’s function distribution leading to dark spot at the center. However, since we have kept pitch length of NCS 0.5λ_spp_, under LCP light it produces bright spot (as shown in Fig. S2(d,e)) as per the following equation:

5 $${E}_{z}(\rho ,\theta ,z)\propto {e}^{-{k}_{a}z}{\int }_{\pi /2}^{3\pi /2}{e}^{j\theta ^{\prime} }\,\cos ({k}_{spp}.{\rm{\rho }}\,\cos (\theta -\theta ^{\prime} )){d}\theta ^{\prime} $$

Therefore, we spot some intensity at the center of NCS-HSPL even though it is zero for other HSPL and standalone SPL.

NCS exhibits linear dichroism, meaning it produces different electric field’s intensity distribution under x and y-polarized light. Under x-polarized light it produces bright spot whereas under y-polarized light it produces dark spot at the center as shown in Fig. S3. Therefore, in case of y-polarized light only SPL contributes to the intensity profile obtained at the center of the hybrid lenses and thus, we have observed almost equal intensity bright spot in the near-field emission at the center of all four plasmonic lenses as shown in Fig. S4.

**Figure 6:**
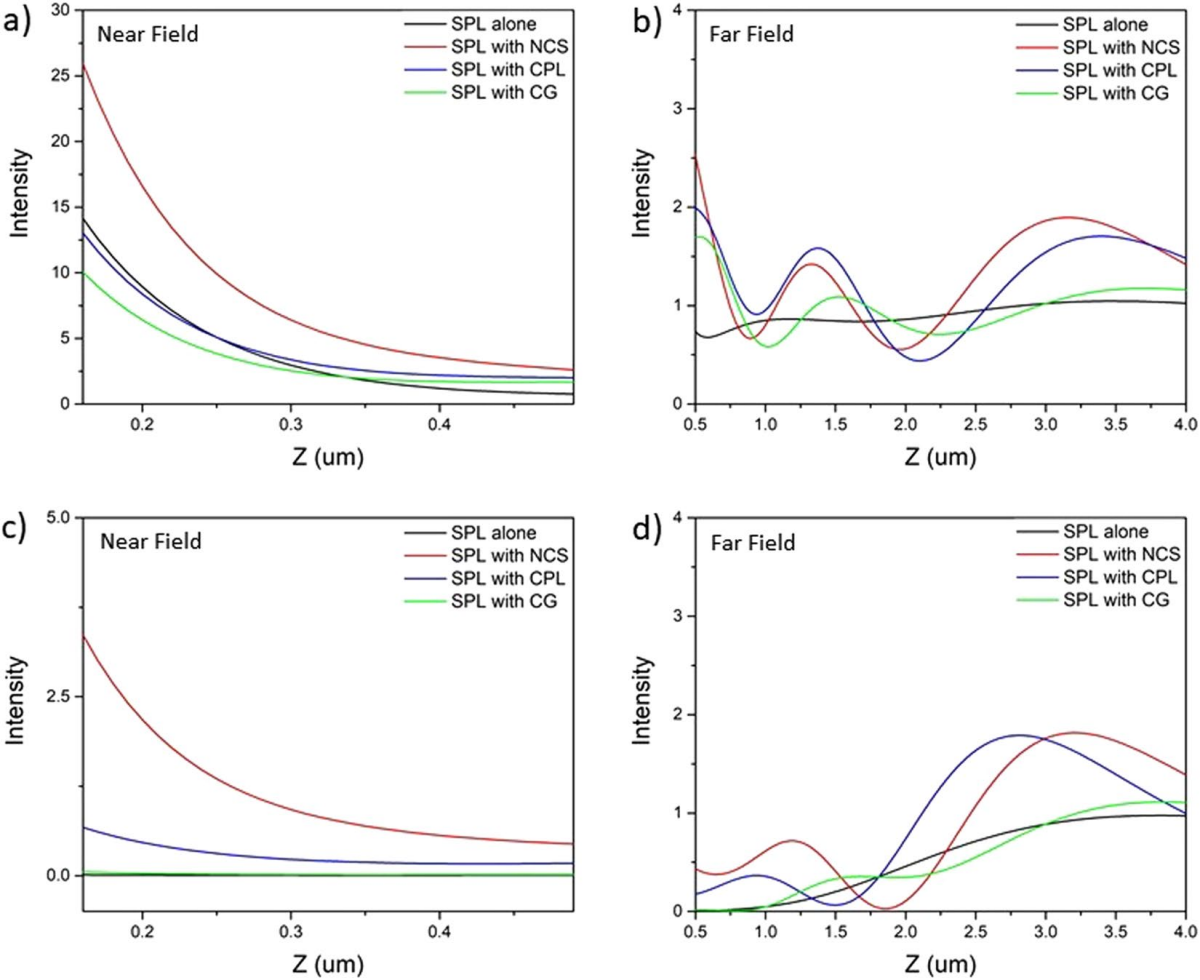
FDTD simulation results of near field and far field intensity distribution on the optical axis for various hybrid lenses. (**a**,**b**) Corresponds to RCP illumination and (**c**,**d**) corresponds to LCP illumination.

From the above discussion, it can be concluded that NCS-HSPL is best among other HSPL in the near-field, however primary objective of using these hybrid lenses is to diffract the propagating SPPs in the far-field. So, in the following section we have evaluated the performances of these HSPLs in the far-field i.e. away from the exit surface of the lens upon illumination with RCP, LCP, x-polarized and y-polarized light. Figure 6 shows comparative study of three different hybrid spiral plasmonic lenses i.e. NCS, CPL, CG inscribed HSPL with simple SPL under RCP and LCP light. It is evident that all the three diffractors create “zones” of maximum intensity in the far-field which is greater than conventional spiral plasmonic lens. Comparing all the three lenses it can be seen that our novel design of hybrid spiral having circular slit and nano corral slit (NCS) clearly demonstrate significantly better local maxima than circular grating which was previously reported^21^ for both left and right circularly polarized illumination. Moreover, HSPL with circular grating reported in the literature has shown reduced intensity in the near field which hinders the utilization of these lenses as the focal length extension has been obtained at the expense of bright intensity spot in near field. Under LCP illumination NCS-HSPL produces bright intensity spot in the near-field whereas other hybrid lenses and standalone SPL exhibit zero intensity at the center as shown in Fig. 6(c). Therefore, under LCP light NCS diffractor can work efficiently in both near-field as well as far-field. So our novel design of hybrid lens having circular slit and nano corrals slit clearly shows the advantage over the reported HSPL. Nevertheless, from Fig. 6a it is apparent that HSPL inscribed with CPL also constrict the near-field intensity. On the other hand, NCS-HSPL not only diffract total field intensity into the far field but also greatly enhances the intensity spot in the near field for both RCP and LCP light.

**Figure 7:**
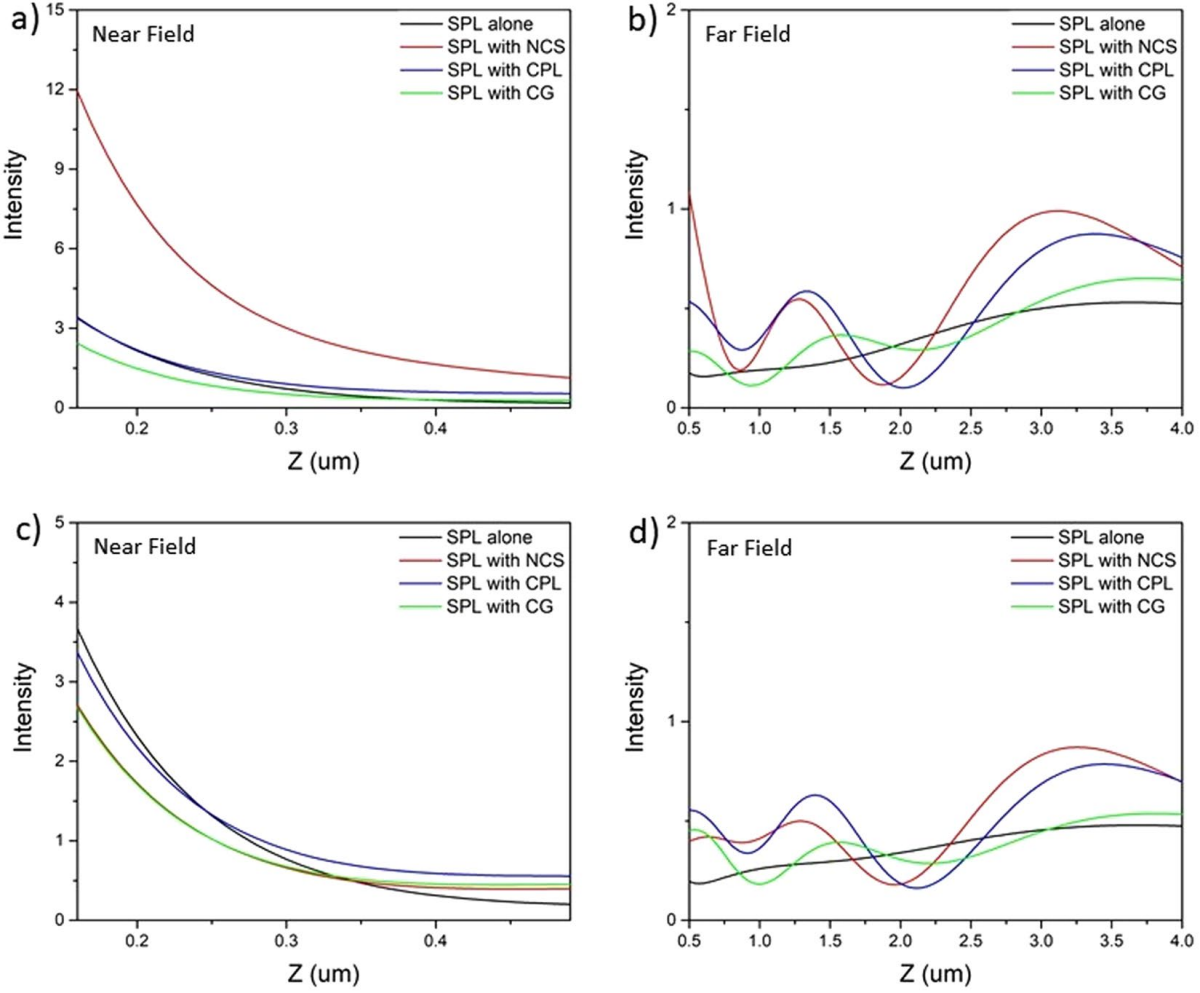
FDTD simulation results of near field and far field intensity distribution on the optical axis for various hybrid lenses. (**a**,**b**) Corresponds to x-polarized light and (**c**,**d**) corresponds to y-polarized light.

**Figure 8:**
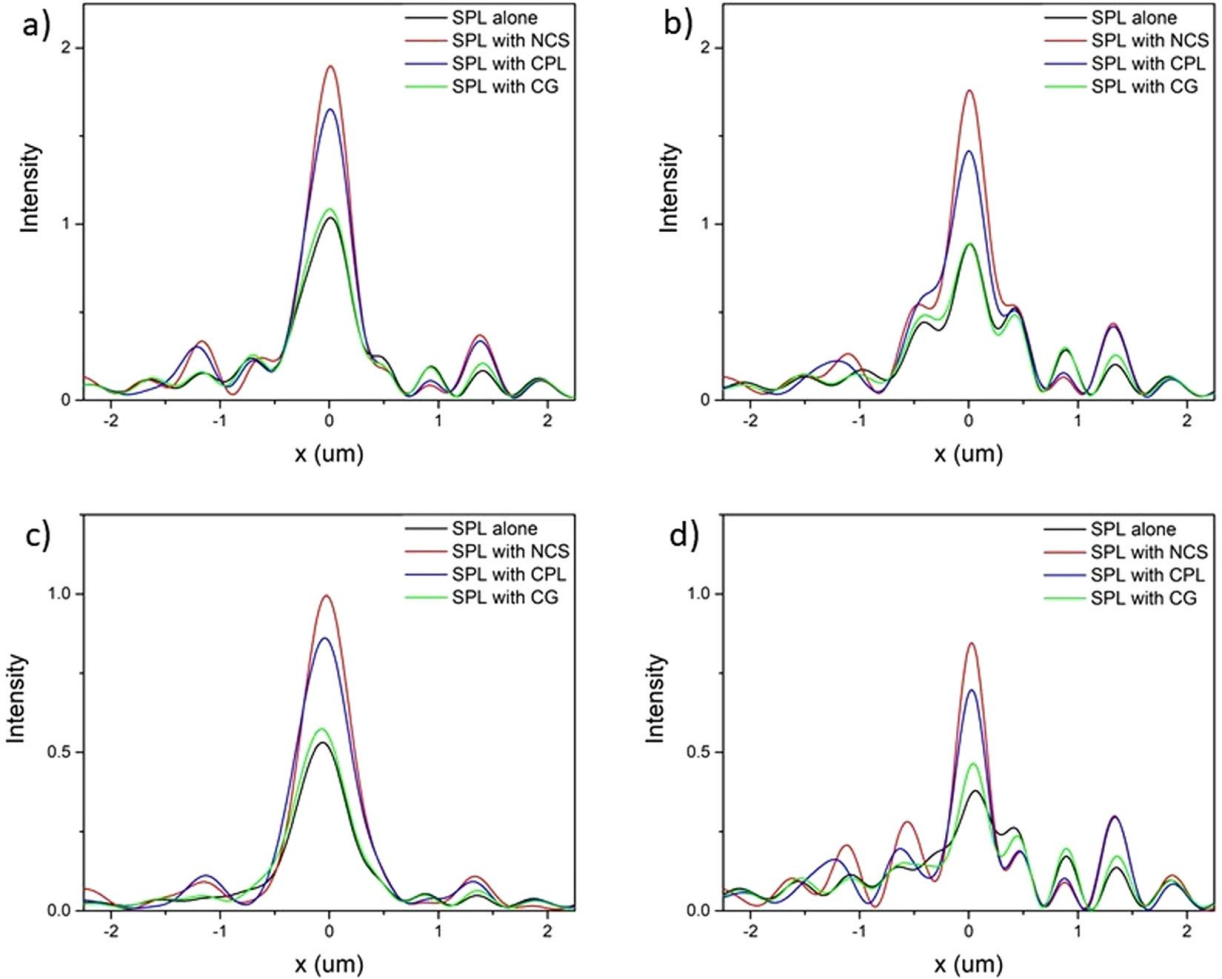
FDTD simulation results of electric field’s intensities (x-direction) of a conventional SPL and hybrid lens under (**a**) RCP, (**b**) LCP, (**c**) x-polarized and (**d**) y-polarized at 3 um above the surface.

Further, we simulated electric field intensity in far field under x and y-polarized light. Figure 7(a–d) shows comparative study of three different HSPL with conventional SPL under x and y-polarized light respectively. Under y-polarized light all lenses have comparable intensity in the near field but in far-field NCS-HSPL dominates as shown in Fig. 7(c,d). After comparing all results from Figs 6 and 7 we can confirm that NCS diffractor is the best for far-field measurements under all types of polarized light. CPL diffractor also competes very well with NCS and completely outperforms previously reported CG in both near field as well as far field. NCS-HSPL shows maximum intensity in the far field approximately at 3 um above the surface of HSPL irrespective of polarization of light. The full width at half-maximum (FWHM) of the focal spot at 3 um above the HSPL surface is shown in Fig. 8. The FWHM in case of NCS-HSPL under RCP illumination has approximately equal size (450 nm) as of SPL even though the light intensity of the center beam spot increases manifold. Therefore, it can be concluded that scattering by the hybrid lens has no influence on the focal spot size of the plasmonic lens. It is also evident from the Fig. 8 that irrespective of the polarization of the light, intensity maxima produced by NCS diffractor is almost twice the magnitude of the one produced by SPL alone. Focal length of 3 um is a sufficient working distance for various plasmonic devices like next generation microscope, sensors, photodetectors, microfluidic lab-on-chip etc.

**Figure 9:**
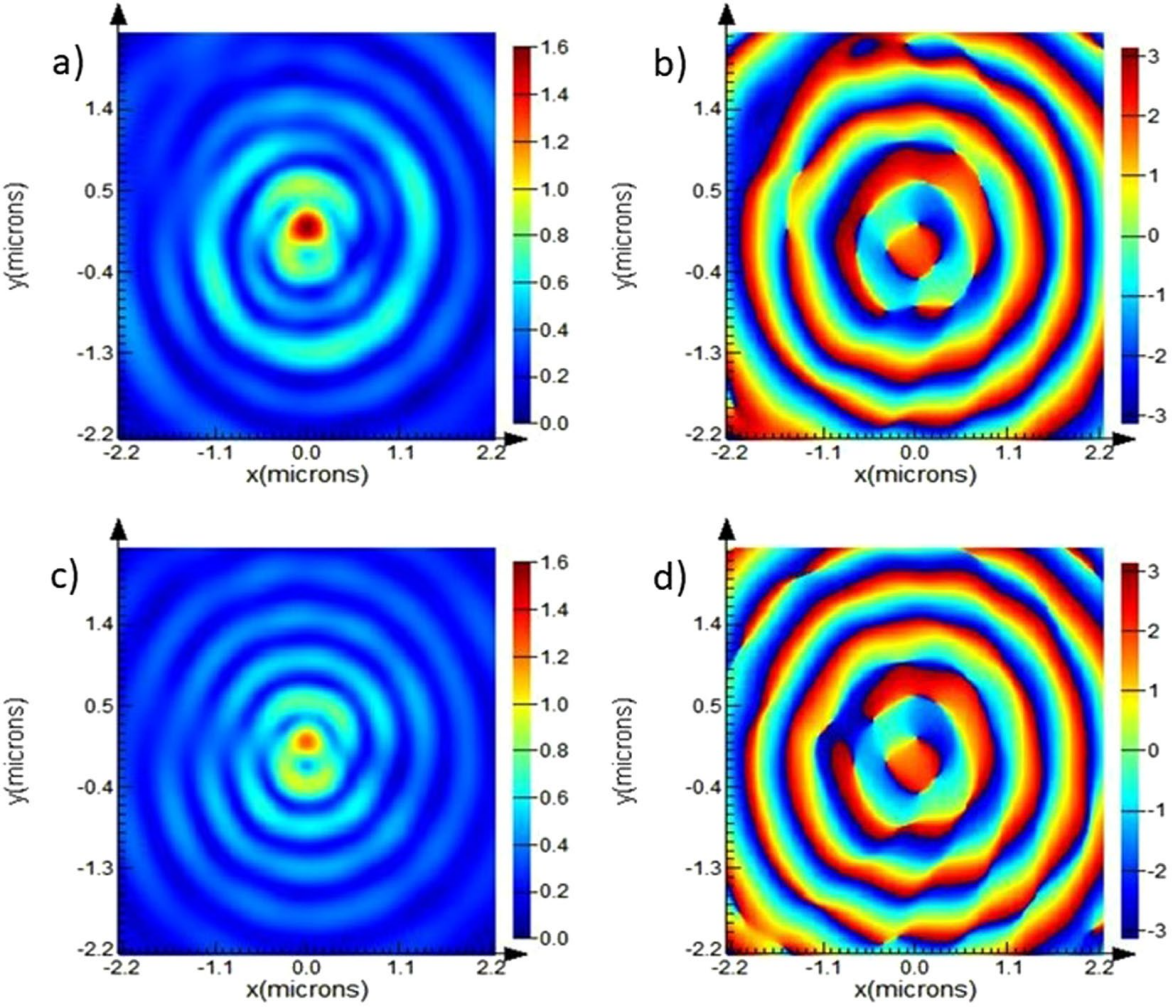
FDTD simulation of electric field intensity and phase distribution of NCS (**a**,**b**) and SPL (**c**,**d**) diffractor under RCP illumination at 3 um above the surface of lens.

It is generally observed that although hybrid lenses produce bright focal spot in the far field, the phase distribution of the SPP-coupled emission often get disturbed. To check the phase distribution of our best performed NCS-HSPL we have compared it with standalone SPL in the far field. Far field distribution of electric field’s intensity and phase for NCS diffractor and SPL at 3 um distance from the exit surface of plasmonic lens are shown in Fig. 9(a–d) respectively. It is evident from the figure that our proposed NCS diffractor has managed to maintain the similar phase distribution at the center of the lens while improving the intensity of the lens.

## Conclusion

In summary, we have conceptualized and designed a hybrid spiral plasmonic lens inscribing NCS and CPL diffractor and compared their focusing properties with previously reported CG diffractor and standalone SPL in both near and far field. We have been able to increase the light intensity in the far field with the use of these diffractor through constructive interference of the SPPs scattered. We have shown that our novel plasmonic structure, NCS-HSPL improves the focal spot in the far field without compromising the intensity in the near field. We have also found that CPL diffractor outperforms CG diffractor irrespective of polarization of light. The far-field light intensity noticeably increases approximately two-fold at 3 um above the surface of the lens owing to the effect of the NCS diffractor for all kind of polarization of incident light. Moreover, the diffractors marginally affect the FWHM of the focal spot even though maximum intensity of the spot increases. Hence, we believe such a lens with long focal length and subwavelength size beam spot will offer a satisfactory working distance for various novel applications such as super resolution microscopy, nano lithography, bioimaging and sensing, optical tweezers, quantum communications, microfluidic lab-on-chip, angular momentum detectors, etc.

## Electronic supplementary material

Supplementary Information
