# Supplementary material for: Far field superfocusing along with enhanced near field emission from hybrid spiral plasmonic lens inscribed with nano corrals slit diffractor
Source: Sci Rep. 2018 Jan 18;8:1127. doi: 10.1038/s41598-018-19571-z (PMC5773565; doi:10.1038/s41598-018-19571-z)
Supplement: Supplementary file 1 — Supplementary Information [file 41598_2018_19571_MOESM1_ESM.pdf]

Far field superfocusing along with enhanced near field emission from hybrid spiral plasmonic lens inscribed with nano corrals slit diffractor

Priyanshu Jain and Tanmoy Maiti*

Plasmonics and Perovskites Laboratory, Department of Materials Science and Engineering,

Indian Institute of Technology Kanpur, UP 208016, India.

Supplementary Note

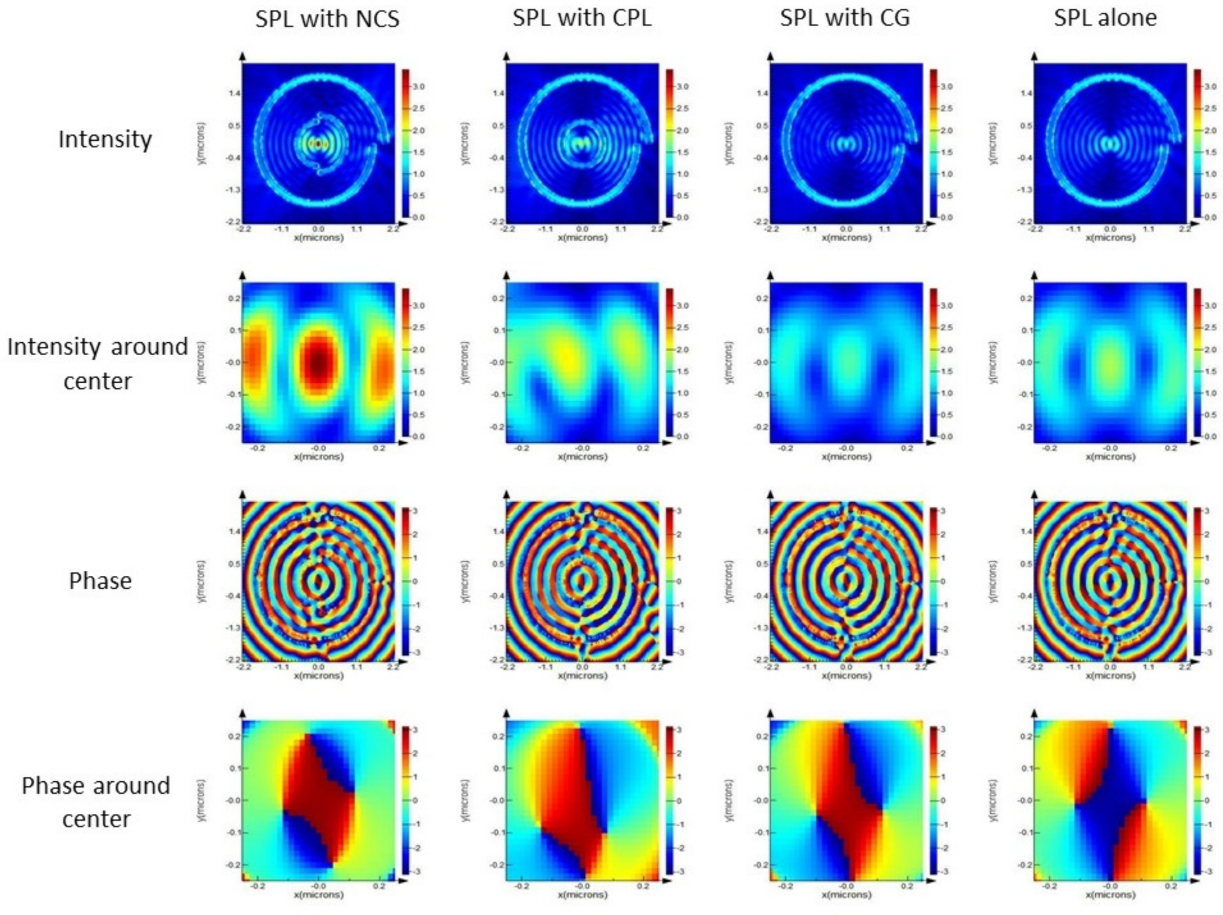

FigureS1 x-polarized light on HSPL: FDTD simulation results of various diffractors inscribed within SPL under x-polarized light in near field. 1st and 3rd row represents intensity and phase profile of lens while 2nd and 4th row represents intensity and phase profile at the center of the lens

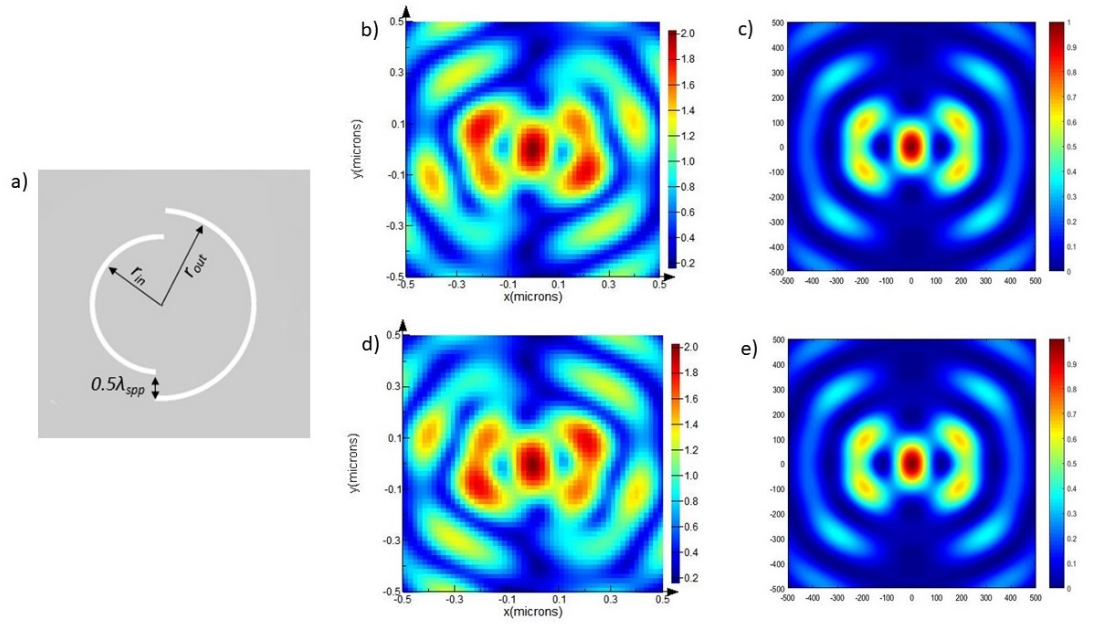

Figure S2. (a) represents the top view of NCS plasmonic lens schematic, (b,d) represents numerical FDTD simulation plots and (c,e) represents corresponding analytical plots of Electric field intensity distribution for the NCS with pitch length, $0.5\lambda_{spp}$. 1st and 2nd row correspond to RCP (σ = -1) and LCP(σ = 1) illumination respectively.

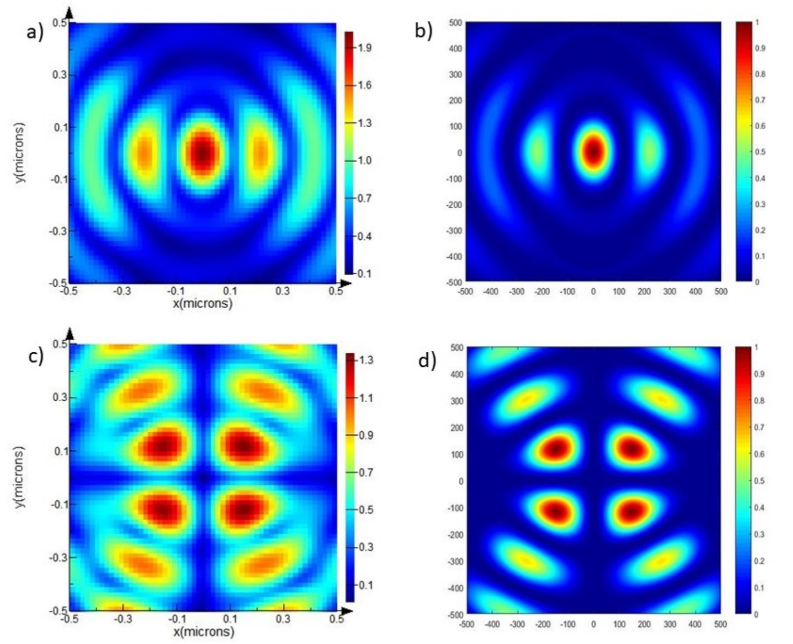

Figure S3. (a,c) represents numerical FDTD simulation plots and (b,d) represents corresponding analytical plots of Electric field intensity distribution for the NCS with pitch length, $0.5\lambda_{spp}$. 1st and 2nd row correspond to x and y-polarized illumination respectively

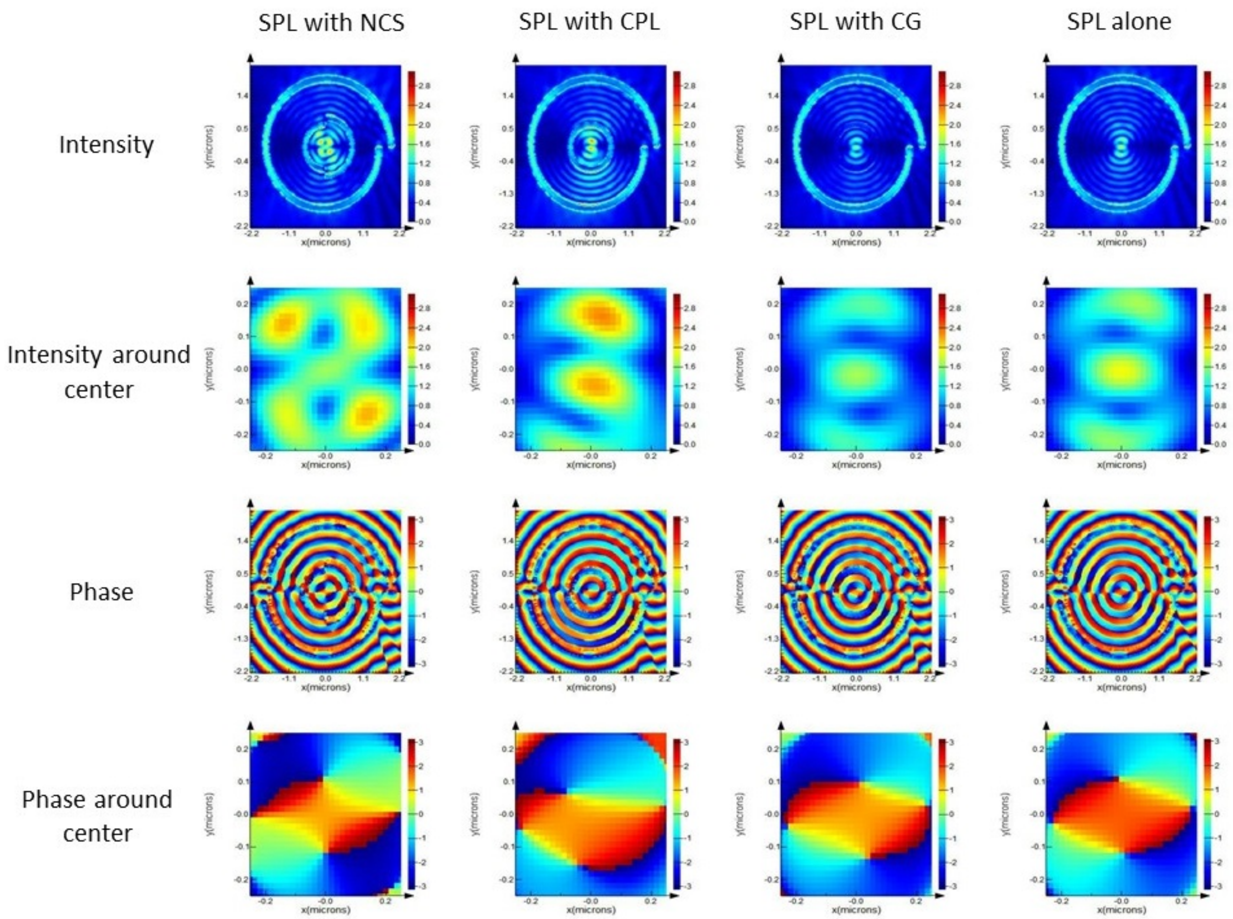

FigureS4 y-polarized light on HSPL: FDTD simulation results of various diffractors inscribed within SPL under y-polarized illumination in near field. 1st and 3rd row represents intensity and phase profile of lens while 2nd and 4th row represents intensity and phase profile at the center of the lens.

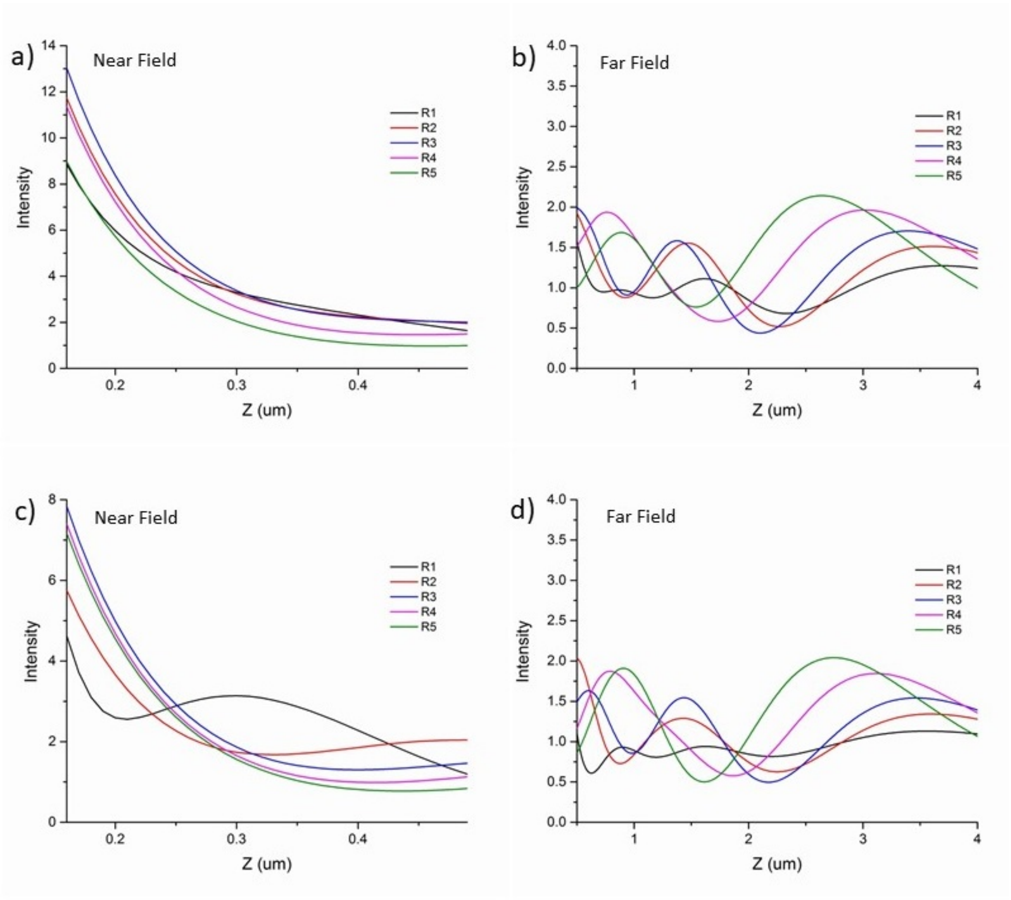

Figure S5. Intensity distributions on the optical axis for hybrid lens with circular plasmonic lens (CPL) under RCP illumination. a) and b) represents CPL with slit radius R1= 0.23, R2=0.43, R3=0.61, R4=0.81 and R5=0.99 um (intensity maxima position) for near and far field respectively. Similarly, c) and d) represents CPL with slit radius R1=0.14, R2=0.34, R3=0.52, R4=0.72 and R5=0.90 um (intensity minima position) for near and far field respectively.

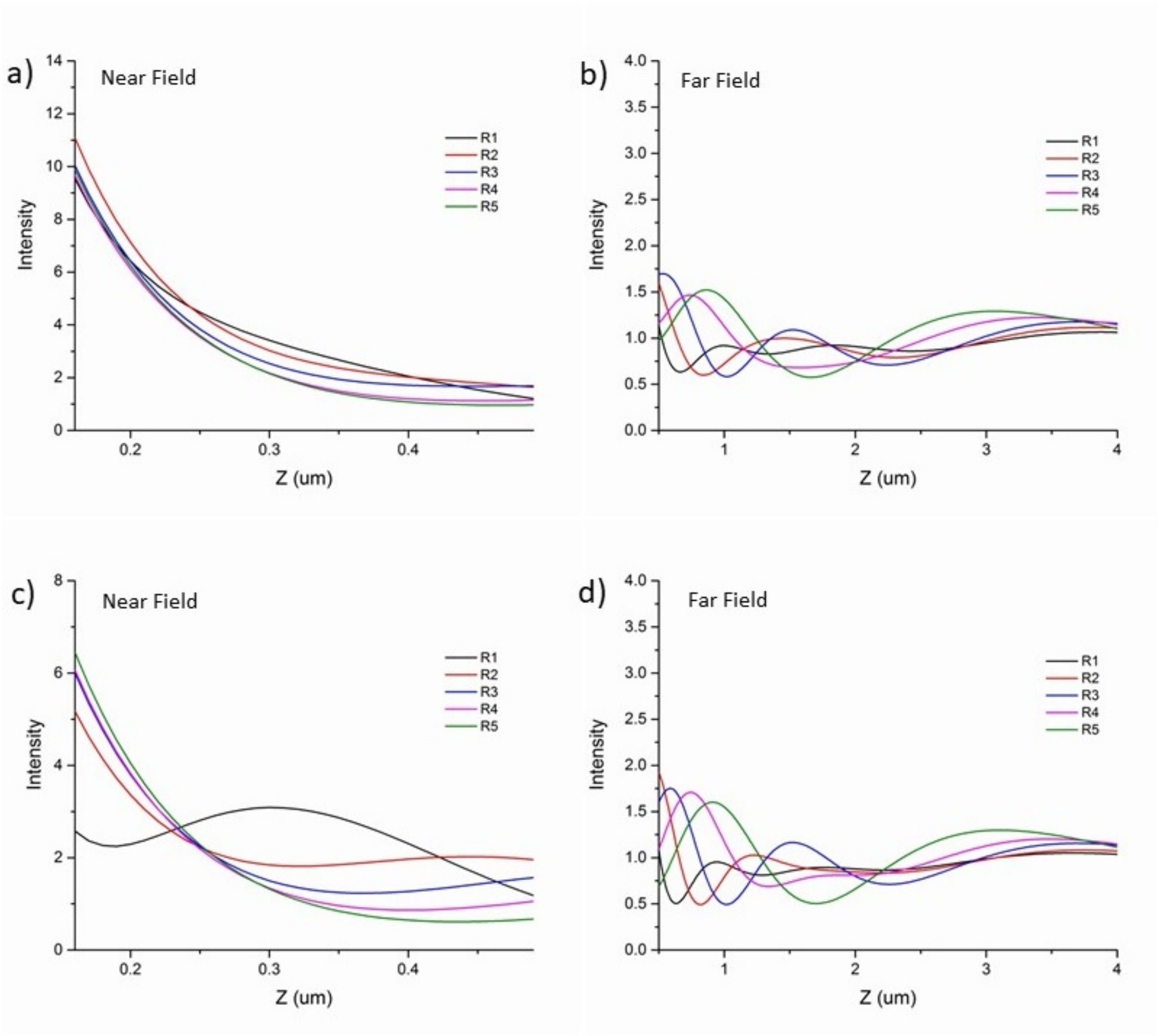

Figure S6. Intensity distributions on the optical axis for hybrid lens with circular grating (CG) under RCP illumination. a) and b) represents CG with slit radius R1= 0.23, R2=0.43, R3=0.61, R4=0.81 and R5=0.99 um (intensity maxima position) for near and far field respectively. Similarly, c) and d) represents CG with slit radius R1=0.14, R2=0.34, R3=0.52, R4=0.72 and R5=0.90 um (intensity minima position) for near and far field respectively.

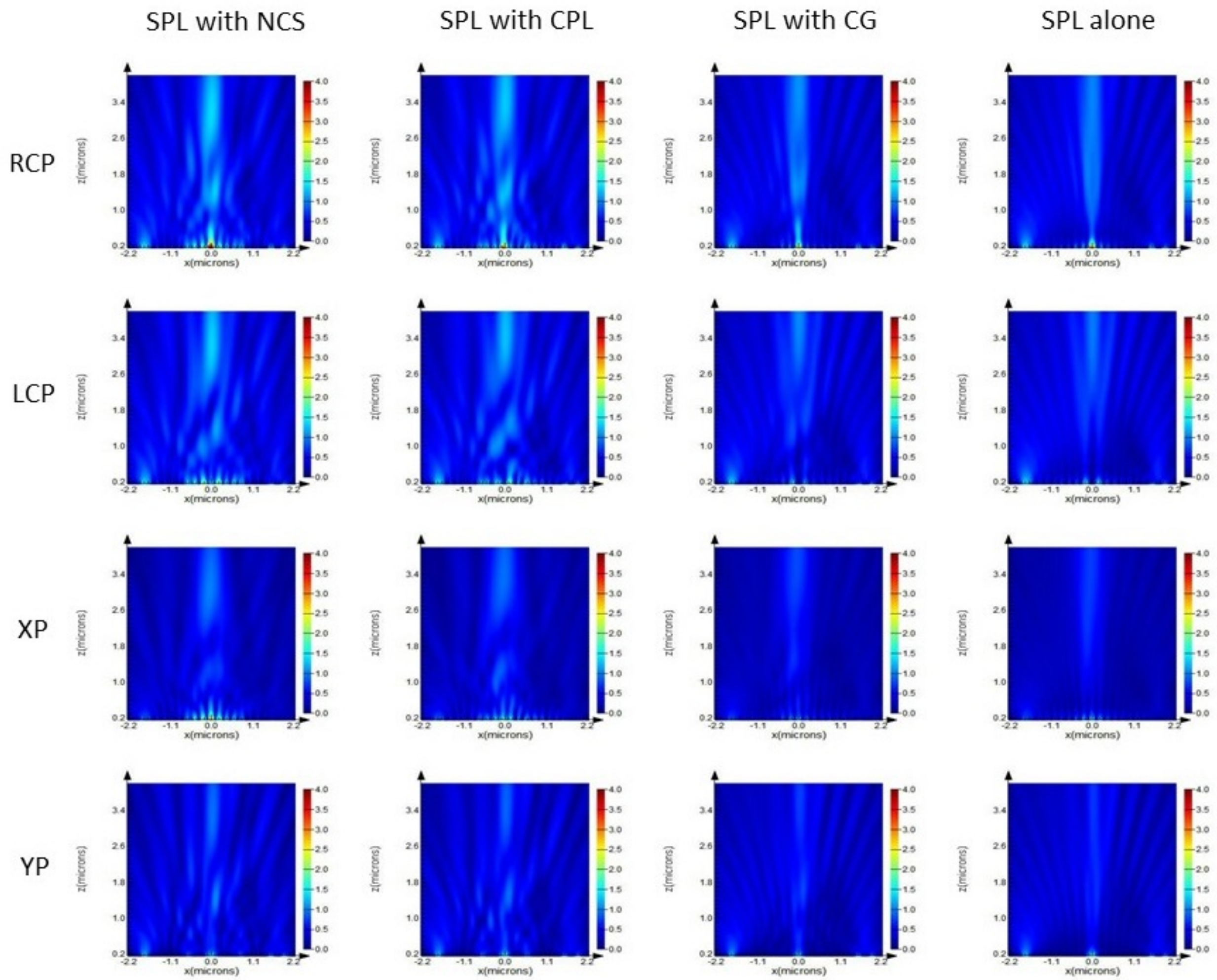

Figure S7. FDTD simulation results of electric field intensity in the far filed along XZ plane for various diffractors inscribed within SPL. First row corresponds to right circular polarization (RCP), second row corresponds to left circular polarization (LCP), third row corresponds to x-polarized light (XP) and fourth row corresponds to y-polarized light (YP).
